# Beyond the algorithm: rethinking the network account of trustworthy ai through lexical threshold-based multidimensional utility analysis

**DOI:** 10.1007/s00146-026-02909-x

**Published:** 2026-03-04

**Authors:** Fei Song, Julian Savulescu, Michael Dunn

**Affiliations:** 1 Centre for Biomedical Ethics, Yong Loo Lin School of Medicine, National University of Singapore, Singapore, Singapore; 2 Uehiro Oxford Institute, University of Oxford, Oxford, United Kingdom

**Keywords:** Trustworthy AI, Network account, Reliability, Beyond technology, Global trustworthiness, Lexical ordering

## Abstract

In this paper, we present an overview of the main conceptual framework for trustworthy AI and argue that the network account offers a superior alternative. We identify and illustrate the possible nodes within an AI network, specify the key attributes each node should possess, and clarify what constitutes a sufficient threshold for each attribute. Building on this foundation, we introduce a novel framework for the network account of trustworthy AI: Lexical Threshold-Based Multidimensional Utility Theory (LTMU). This framework lexically ranks trust-relevant attributes, establishes minimum thresholds for each and assesses each attribute with a distinct utility scale. We then demonstrate how the LTMU framework can be applied to analyse two exceptional cases in which trust-relevant attributes come into conflict in different ways, arguing that its application yields reasonable and defensible outcomes in both scenarios.

**Supplementary Information:**

The online version contains supplementary material available at 10.1007/s00146-026-02909-x.

## Introduction

Trust is a fundamental aspect of human relationships, enabling us to rely on one another despite uncertainty, facilitating planning, cooperation, and coordination. With the emergence of AI technology and its widespread application across nearly every aspect of daily life, a critical question arises regarding the human-AI relationship: Can we trust AI in the same way we trust other people? If the answer is yes, what criteria define a trustworthy AI?

Classical accounts of interpersonal trust assert that trust includes normative expectations about the trustee’s motives or commitments, beyond just expecting reliable performance. Applying this claim to the context of AI raises challenges: if trust requires the trustee to have the correct intentions or responsibilities toward the trustor, can mindless machines qualify as trustees? There are three main positions regarding whether trustworthy AI is conceptually plausible and whether we can trust in AI. The first position, which we refer to as the non-anthropocentric approach, argues that a definition of trustworthiness based on strongly anthropocentric psychological traits—such as goodwill—cannot be generalized to account for trustworthy AI. (Simion and Kelp 2023) This is because today's AI lacks inner psychological attributes like goodwill. Accounts of trust that do not treat psychological traits as a necessary condition for trustworthiness are better suited to explaining what it means for AI to be trustworthy.

The second position contends that AI can only be considered reliable as it lacks autonomy, agency, and the capacity for goodwill. In this view, reliance can be placed on AI, but trustworthiness cannot. Al (2023), for example, draws on the works of philosophers Karen Jones (1996) and Thomas Simpson (2012), and explores the evolution and function of trust in human societies. He highlights that trust involves expectations of goodwill and moral competence; attributes that AI systems do not possess. While animals and institutions can exhibit forms of responsiveness, narrow AI systems operate based on predefined algorithms without understanding or moral awareness, rendering them incapable of genuine trustworthiness. Ryan (2020) holds a similar position and argues that, according to the most widely accepted definition of trust, which incorporates goodwill and agency, AI does not possess the necessary attributes to be trusted. It can only be relied upon. This distinction is crucial to prevent misplaced expectations and to foster a more accurate understanding of human–AI relationships. This position finds popularity, particularly within the field of applied philosophy of technology (see Hatherley 2020; Ryan 2020). One advantage of this position is its alignment with our inherent inclination towards interpersonal and anthropocentric trust relationships.

We believe that a more compelling conceptual framework should include two key components: (a) it should be compatible with non-Western cultural perspectives, and (b) it should broaden the scope of analysis beyond AI algorithms. However, the two most prominent frameworks lack. The recently developed network account of trustworthy AI (Song 2023) is a better alternative as it can satisfy the two components. It offers a promising alternative in how it understands trustworthiness as an emergent property of the entire network of interactions of different nodes—technical, human, and institutional—that make up an AI system. However, this network account requires further conceptual development to clarify how the distinctive and potentially incommensurable attributes of each node jointly determine the overall trustworthiness of an AI system. For example, suppose an AI system is technically reliable, but the human actors involved—such as developers or companies—fail to demonstrate goodwill or ethical responsibility toward users, and there is no robust institutional framework to ensure accountability. Can such a system truly be considered trustworthy? This paper attempts to provide a novel solution to this question.

In this paper, we start with an overview of the main conceptual frameworks for trustworthy AI, focusing on the contraposition between anthropocentric and non-anthropocentric approaches in Sect. 2. In Sect. 3, we argue that these dominant conceptual frameworks are inadequate. In Sect. 4, we specify the key attributes each node should possess and what constitutes a sufficient threshold for each. We further propose a novel framework for the network account of trustworthy AI: Lexical Threshold-Based Multidimensional Utility Theory (hereafter LTMU). This framework clarifies how each of the nodes in the network relates to an overarching determination of trustworthiness, requiring a lexical ranking of trust-relevant attributes, setting minimum thresholds for each, and evaluating them using distinct utility scales. We argue that this method offers a better solution than a weighted sum approach or a lexical ordering approach for assessing the trustworthiness of an AI network when the trust-relevant attributes across one or more nodes are in conflict. We demonstrate how the LTMU framework can be applied to analyse two exceptional cases in which trust-relevant attributes come into conflict in distinct ways. We argue that the implications of the LTMU framework yield reasonable and defensible outcomes in both cases.

## Trustworthy AI: Can We Trust in AI or Merely Rely on AI

One of the theories that is under the non-anthropocentric framework is Simion and Kelp’s obligation-based account (2023). They apply their obligation-based account of trust to the concept of trustworthy AI. They distinguish between design functions (d-functions), which arise from the designer’s intentions, and etiological functions (e-functions), which emerge from a history of successful performance. A subject can form normative expectations of an artificial agent (e.g., AI) based on its function-derived obligations. If an AI has the strong dispositions to fulfil both d-functions and e-functions, it should be considered maximally trustworthy (p.8).

Kelp and Simion’s account of trustworthiness (2023) provides a particularly illuminating and sophisticated framework that sharpens the distinction between mere reliability and genuine trustworthiness. Their central claim—that trustworthiness is best understood as a disposition to fulfil one’s obligations—offers a remarkably clear way of capturing what is normatively significant about being trustworthy. One of the strengths of their view is that it preserves the conceptual difference between trustworthiness and reliability while still acknowledging the important, but non-essential, role of goodwill. As they convincingly argue, goodwill is not required for trustworthiness; instead, it enhances or intensifies it (p. 678). Still, however, even on their minimalist account, the notion of obligation—understood in functional or role-based terms—appears to presuppose a degree of agency that may not extend to all entities (e.g. AI) under consideration. Carter (2023) raises concerns about whether this obligations-based account can apply to artificial agents. He questions whether AI, which lacks consciousness and intentionality, can hold obligations in a manner analogous to human agents. (p. 18). Put another way, Kelp and Simion’s account may successfully distinguish reliability from trustworthiness, but it remains insufficient to fully capture the notion of trustworthy AI, as contemporary AI systems fail to meet even the minimal requirements of genuine agency.

However, a non-anthropocentric perspective on trustworthiness presents certain drawbacks. First, an approach that is less centred on human perspectives often fails to distinguish between mere reliance and trust, and the consequence is that it often yields unrealistic implications when applied to artefacts. For instance, we typically do not experience a sense of trust towards our cars or smartphones. As O'Neill (2002) argues that when we claim that “I trust my cars or smartphones”, it involves misplacing trust in someone or something that does not warrant it. Tavani (2015) contends that we only experience disappointment when an AI fails to act in accordance with our interests, but we do not feel betrayed by the system. This is because, conceptually speaking, only fully moral agents, possessing autonomy and agency, are capable of betraying our trust.

The second approach, which claims that AI is merely reliable, also faces challenges. If AI can never meet the psychological criteria for trust—such as the capacity for goodwill—then establishing a justified trust relationship between humans and AI becomes unattainable. This poses a significant challenge in domains where trust is essential, such as in medicine, finance, and education.^1^

A third set of positions seek to take a middle ground, emphasising the importance of distinguishing interpersonal trust from trust in technology. Some scholars propose that, while the conceptual core of trust (reliance plus some extra factors) remains, trust in AI should be treated as a distinct notion separate from interpersonal trust​. Tavani (2015), for example, claims that genuine trust relationships can exist between humans and artificial agents (hereafter AA), but understanding this requires a multi-level approach​. He employs James Moor's four-level model of ethical agency^2^ to construct a nuanced framework for analysing trust in H-AI interactions. Tavani (2015) proposes that four different levels of trust correspond to the agent's different ethical agency. The different levels of trusts are (1) Trust in Ethical Impact Agents: Predicated on predictable behaviour and reliability, without attributing moral understanding to the agent. (2) Trust in Implicit Ethical Agents: Based on the system's design features that aim to prevent harm, fostering a form of trust rooted in safety mechanisms. (3) Trust in Explicit Ethical Agents: Involves confidence in the agent's ability to process and apply ethical principles, suggesting a higher degree of trust due to the agent's decision-making framework. (4) Trust in Full Ethical Agents: Encompasses a profound level of trust akin to that between humans, contingent on the agent possessing consciousness and intentionality.

According to this four-level account, trusting a basic automated tool with no ethical reasoning (such as a thermostat to regulate temperature) is not the same as trusting a highly autonomous AI that makes explicit moral decisions (such as a self-driving car or a robotic caregiver). Tavani contends that trust evolves from simple reliability-based trust at lower levels to more robust, interpersonal-like trust at higher levels, paralleling the agent’s capacity for ethical action.

Giacomo Zanotti et al. (2024) offer a different proposal within this third position. They argue that human–AI trust—corresponding to levels 1–3 in Tavani’s framework (i.e., trust in ethical impact agents; trust in implicit ethical agents; and trust in explicit ethical agents) —should not only be understood as a distinct kind of trust, but should also be assessed according to criteria different from those used for interpersonal trust (level 4 in Tavani’s framework—trust in full ethical agents). They suggest that both forms of trust require the trustee to be reliable, but Human–AI trust differs in that it does not presuppose goodwill or moral obligation, as interpersonal trust does. Instead, it demands additional ethical dimensions, such as fairness, transparency, data privacy protection, explicability, accountability, and respect for human autonomy (Zanotti et al. 2024, p. 2696).

The major critique of the third position is that a distinct notion of “trust” in AI is based on the assumption that if AI fills a role normally occupied by a human trustee, it automatically enters a trust relationship. But this is questionable. Fossa (2019), for example, ​argues that although humans may routinely behave as if they 'trust' a well-functioning AI, such trust may be misplaced. He calls Tavani’s level-based approach “thought-provoking” but “too coarse-grained,” cautioning that it glosses over crucial differences between trusting persons and trusting artifacts. (p. 68) A similar challenge may also be raised against Zanotti et al.’s account, although their proposal is more effective in distinguishing trust in AI from mere reliance on it. This is because trust, as they conceptualize it, involves holding the AI system to meet particular ethical standards—such as fairness, transparency, explicability, data privacy, and respect for human autonomy. Nonetheless, one issue with their approach lies in the ethical attributes they emphasize—such as accountability, privacy, and data governance—which are presumably not realized by AI technologies (e.g., algorithms), but rather by human agents.

## Current frameworks are inadequate

One important feature of the overview of the current main literature that we have provided above is that the three main frameworks for trustworthy AI are exclusive to different extents. The exclusivity manifests in two main keyways: (1) the conceptual analysis is overly shaped by a Western cultural perspective; and (2) the assessment is narrowly focused on AI algorithms, neglecting other dimensions in an AI system.

### Trustworthiness is a thick ethical attribute in confucianism tradition

The arguments advocating for expanding the notion of trust to accommodate artifacts like AI, this inclination is largely shaped by Western philosophical and linguistic traditions. In contrast, Confucian perspectives on trust—embodied in the Chinese term xìn (信)—present significant concerns with expanding trust in this way.

In Confucian thought, *xìn* is one of the "five constant virtues" and is deeply anthropocentric. According to Sung (2020), in some passages, *xin* is discussed in relation to speech (e.g., *Analects* 5.10, 15.5, 7.25). Cecilia Wee (2011) further argues that *xin* is primarily concerned with verbal commitments. Importantly, *xìn* is not simply about keeping promises—it is context-sensitive (*Analects*, 13.20, *Mencius*, 9.39) and ethically complex*.* (*Analects* 1.4, 1.7, 1.8, 5.25, 9.25, 5.28, 7.25, 9.25, 12.10, 15.6. *Mengzi* 4A:12 and 5A:12) A trustworthy person in the Confucian tradition must evaluate situations to determine whether fulfilling a prior commitment remains ethically appropriate. For instance, if one had promised to help a friend but later discovered the friend committed murder, withholding assistance would be seen as the morally right decision. Trustworthiness, in this framework, involves ethical discernment, not rigid rule-following.

This "thick" moral conception of trust complicates attempts to ascribe trustworthiness to AI. To be regarded as trustworthy in the Confucian sense, AI would need more than reliable performance—it would require ethical capacities akin to sincerity, contextual judgment, and moral integration with other virtues. While large language models (LLMs) might mimic verbal commitments, genuine *xìn* requires that such communication be rooted in internal ethical understanding and commitment.

### Beyond AI technology

Theories across all three main frameworks primarily concentrate on AI technologies—particularly the performance of AI algorithms, such as whether they are reliable, fair, transparent or explainable. Zanotti et al.'s proposal suggests that the concept of trustworthy AI should go beyond purely epistemic requirements, such as reliability and robustness. However, they do not explicitly clarify whether the additional ethical requirements—such as accountability, transparency, and fairness—are intended solely for AI algorithms or also extend to other components, such as AI companies or socio-legal institutions.^3^

The concept of trustworthy AI extends beyond the technology itself, while not entirely new, has yet to be widely discussed or adopted as a prominent perspective. Lahusen et al. (2024) recently suggest that ensuring the trustworthiness of AI governance requires addressing machines, humans, and institutions at the same time*.* For instance, a city using an AI system to control traffic flow must ensure the system is technically reliable (no dangerous failures), and that policies are in place to prevent abuses (like not unfairly prioritizing certain districts). Public consultations require transparency about how AI is used in governance, and avenues for redress can all help build citizens’ trust.

## A Network account of trustworthy AI: nodes and assessment

Song’s network account of trustworthy AI is a promising alternative to the dominant frameworks currently in use. This account holds significant potential for accommodating various conceptions of trust—both “thin” accounts (e.g., obligation-based) and “thick” accounts (e.g., goodwill-based)—as well as culturally distinct concepts of trusts, such as the Confucianism concept of trust. According to this account, if we adopt the goodwill-based account of trust, the network perspective may suggest that while AI technology itself can only be reliable due to its lack of moral agency, the broader AI network can still be trustworthy—provided that AI companies act responsibly, professionals uphold ethical standards, and robust socio-legal systems are in place. In contrast, if we adopt a weak or “thin” conception of trustworthiness—such as the functional obligation-based account—the network view might imply that AI technology could itself be considered trustworthy. However, this alone is insufficient. A genuinely trustworthy AI system also requires trustworthy and ethical attributes from AI companies and professionals, as well as well-established legal and social systems.

One concern with a network-based account of trustworthy AI, however, is the lack of a clear method or mechanism for assessing the trustworthiness of an entire AI network. A typical AI network may consist of distinct nodes, each potentially requiring qualitatively distinct attributes to contribute to the trustworthiness of the network. This poses significant challenges for quantifying these attributes on a single scale, making comparison difficult and complicating efforts to balance or trade off conflicting attributes. Without a sound method for global assessment, the plausibility of the network framework is weakened, potentially leading to moral paralysis in decision-making.

In this paper, we further develop the network account of trustworthy AI by pursuing two main objectives: (1) to provide a more detailed account of the possible nodes within an AI network and the specific attributes each should possess in a trustworthy system; and (2) to propose a novel method for the global assessment of an AI system’s trustworthiness, in particular, when some attributes are in conflict.

### Four distinct nodes: technology, developers, professionals and sociolegal systems (TDPS)^4^

AI systems can significantly depend on the functions they serve and the domains in which they are applied. As a result, different AI technologies may involve distinct types of nodes. However, there are four general categories of nodes that should be included in any AI system, though we remain open to considering additional types beyond these four^5^:

*AI Technology* There are at least three main approaches to developing AI software. The first approach adopts the rule-based system (symbolic AI), which operates through explicit knowledge representations (e.g., logical rule, knowledge graphs) and deterministic reasoning. (Grosan and Abarham 2011; Xu et. Al. 2023) These systems exhibit inherent transparency as the inferential chain of rules and symbolic manipulations leading to a conclusion that can be explicitly traced, which thereby facilitates human interpretability and systematic auditability. However, symbolic programs typically handle noise and uncertainty poorly: if an input falls outside the predefined rules, a symbolic system often fails to respond robustly.

The second approach is the neural networks approach (deep learning systems), which operates by being trained on large datasets, generalising from big data to make accurate predictions or classifications on new data. (Mohsen et. Al. 2023; Sarker 2021) Pure deep learning models, while powerful, often generalise poorly beyond their training distribution. Meanwhile, the decision processes are encoded in millions of weights, making it difficult to trace how a specific output was derived—which is often known as the “black box” problem. This opaqueness undermines explainability and can mask unsafe behaviour—for example, large language models may “hallucinate” false facts or produce harmful recommendations with no obvious warning, since they have no built-in understanding of truth or ethical rules.

The third approach is the neuro-symbolic approach (hybrid system), which combines the rule-based and deep learning systems, mitigating the issues of the two approaches. The core design is to enforce logical constraints or domain knowledge alongside statistical learning from big data, so that neural network outputs are guided by symbolic reasoning and expert-defined rules.^6^ Neuro-symbolic systems are thought to improve reliability and explainability by combining the complementary advantages of neural and symbolic methods, thus yielding more robust and transparent outcomes. (Hitzler et al. 2022).

These three approaches, although distinct from one another, share a common objective: the development of a reliable AI system. But how, precisely, should we assess whether an AI system is reliable? Grote et al. (2024) provide a clear and comprehensive review of reliability in machine learning. According to their overview, there are at least four approaches to assess the reliability of AI reliability. They are (a) statistical learning theory;^7^ (b) robustness under shifting conditions;^8^(c) socio-technical embedding of models among human decision-makers,^9^and d) the opacity of models’ internal reasoning.^10^Building on this, we offer three suggestions for ensuring the reliability of AI technology, as well as criteria for what can be considered sufficient for a reliable AI software system.

First, an ideally maximally reliable AI model should meet the following criteria: (1) high predictive accuracy within the data distribution; (2) sustained high accuracy under different conditions or across varied data distributions (e.g., a model trained on early COVID-19 data from 2020 should be able to accurately predict symptoms of COVID-19 infection in 2025); (3) the results must be endorsed by domain experts and trusted by the public; and (4) the model's internal reasoning should be explainable.

Second, reliability is not a one-time checklist, but an ongoing, iterative process aimed at maintaining the model's performance within acceptable levels. Given the extreme difficulty of achieving a maximally reliable model,^11^we suggest that, rather than insisting on perfection, we should accept a model that meets the reliability criteria at an acceptable level.

What is considered "acceptable" or "reasonable" may vary depending on the context. For example, slightly lower predictive accuracy or robustness might be acceptable in a low-stakes application, such as CV selection in job hiring, but not in a high-stakes domain, such as medical diagnosis or finances. Furthermore, in some contexts (e.g., medical diagnosis), a higher level of explainability^12^ and interpretability maybe required than in other contexts (e.g., stocks).

*AI Companies or developers* The organizations or individuals who create and maintain the AI systems. The well-known AI companies or developers are OpenAI, which is known for developing advanced LLMs models like-GPT; DeepMind is renowned for breakthroughs, such as AlphaGo and AlphaFold, and is heavily invested in AI research for healthcare, gaming, and more.

The trustworthiness of AI companies lies in the conduct of the given organization that how they develop and deploy the technology. We propose that we should assess the trustworthiness of AI companies in at least in the following aspects: (1) transparency; (2) fairness; and (3) regulatory compliance.^13^

Transparency is often the most important attribute of a trustworthy organization. In the context of AI, it refers to openness about AI systems’ design, training, and limitations. In practice, however, AI companies vary greatly in the level of transparency.^14^ Moreover, transparency is not only about model architecture but also is about how data is governed. For example, OpenAI provides a *“*Trust Portal*”* outlining its privacy and security policies for enterprise users.^15^ To demonstrate the transparency attribute, companies should regularly publish reports in detail on their model structure, as well as how they protect and govern data. The reports must not only be comprehensive but also evidence based—key and substantial evidence should be provided^16^.

Fairness is the second important attribute of a trustworthy AI organization. Large AI models have demonstrated biases (gender, racial, political, etc.) learned from training data. AI companies, such as OpenAI, have acknowledged biases in GPT-3 and subsequent models, and have taken steps, such as refining training datasets, filtering out toxic content, and incorporating user feedback loops to *“*minimize harmful outputs.”^17^

Finally, whether AI companies comply with AI regulation is another attribute we should assess. Regulatory compliance refers to AI organizations adhering to existing laws (like data protection, consumer protection, non-discrimination laws) and preparing for upcoming AI-specific regulations. In the European Union, for example, the EU *Artificial Intelligence Act*, the world’s first comprehensive AI regulation, introduces requirements on transparency, risk assessments, and even possible restrictions on general-purpose AI systems. (European Union, 2024)^18^ The assessment of an AI company's trustworthiness should focus on how well it complies with those regulations.^19^

*Professionals who use AI technology* In professional domains from healthcare to finance, AI systems are increasingly acting as collaborators alongside human experts. This human–AI teamwork holds great promise for improved decision-making and efficiency. The trustworthiness of the experts who use AI technology becomes important. One of key requirements for trustworthy experts is their competence of assessing and weighing AI-generated predictions. If professionals place *too much* trust in AI, they might accept AI recommendations uncritically, leading to errors being overlooked. In contrast, if they trust the AI *too little*, they may ignore useful advice, missing its benefits, failing to prevent harm and reducing the efficiency. (Lebiere et al. 2021).

In practice, the primary challenge is that the black-box nature of AI algorithms makes it difficult for experts to understand how outputs are generated, and consequently, to assess their accuracy. Explainable AI can help mitigate this uncertainty by providing insights into the reasoning behind the outputs. (Cheung & Ho 2025) Nevertheless, the quality of explanations is strongly correlated with the level of trust users place in the system. In some cases, overly complex or poor explanations diminished trust (Rosenbacke et al. 2024).

Since current explainable AI is imperfect and the quality of explanations varies, trustworthy professionals should also continuously monitor and question the AI’s performance. In medical practice, this means a doctor should question an AI’s surprising or counterintuitive diagnosis, seek for reasonable explanations and make a judgment if the diagnosis is correct.^20^

*Social-legal systems for addressing AI risks and harm* Frameworks or institutions that address, manage, and regulate the risks and harms associated with AI are necessary for a trustworthy AI network. A robust social-legal system should at least have two features: first, it should assign responsibility to the organizations that develop and deploy AI systems; and second it should extend to the professionals who use AI within specific domains. In many traditional jurisdictions, manufacturers are strictly liable for injuries caused by defective products. It means that if an AI system or robot is considered a product, liability may arise under product liability law without needing to prove negligence of the manufacturers. For instance, if a self-driving car’s software is defectively designed and causes a crash, the victim could sue the car manufacturer under strict product liability rather than trying to prove the company was careless.

In high-stakes fields like medicine and finance, when the H-AI team framework is applied, professional bodies should also hold accountability. Doctors cannot abdicate liability and accountability to an AI predications or recommendations—instead they must understand the AI’s limits and verify its suggestions, weighing different values and make an all-things-considered decision. If they failed to do so, they are accountable or strictly liable for harm that patients suffer.

Although the opacity and complexity of AI systems often make it difficult to assign responsibility to a particular agent, it is reasonable to presume that AI companies, model developers, manufacturers, and domain professionals should bear shared strict liability when harm or bias arises from the human–AI deployment loop. This idea is analogous to the concept of market-share strict liability in tort law, which assigns legal responsibility to all manufacturers of a defective product based on their respective shares of the market at the time the harm occurred—especially when it is impossible to identify which specific manufacturer caused the injury.^21^

**Fig. 1:**
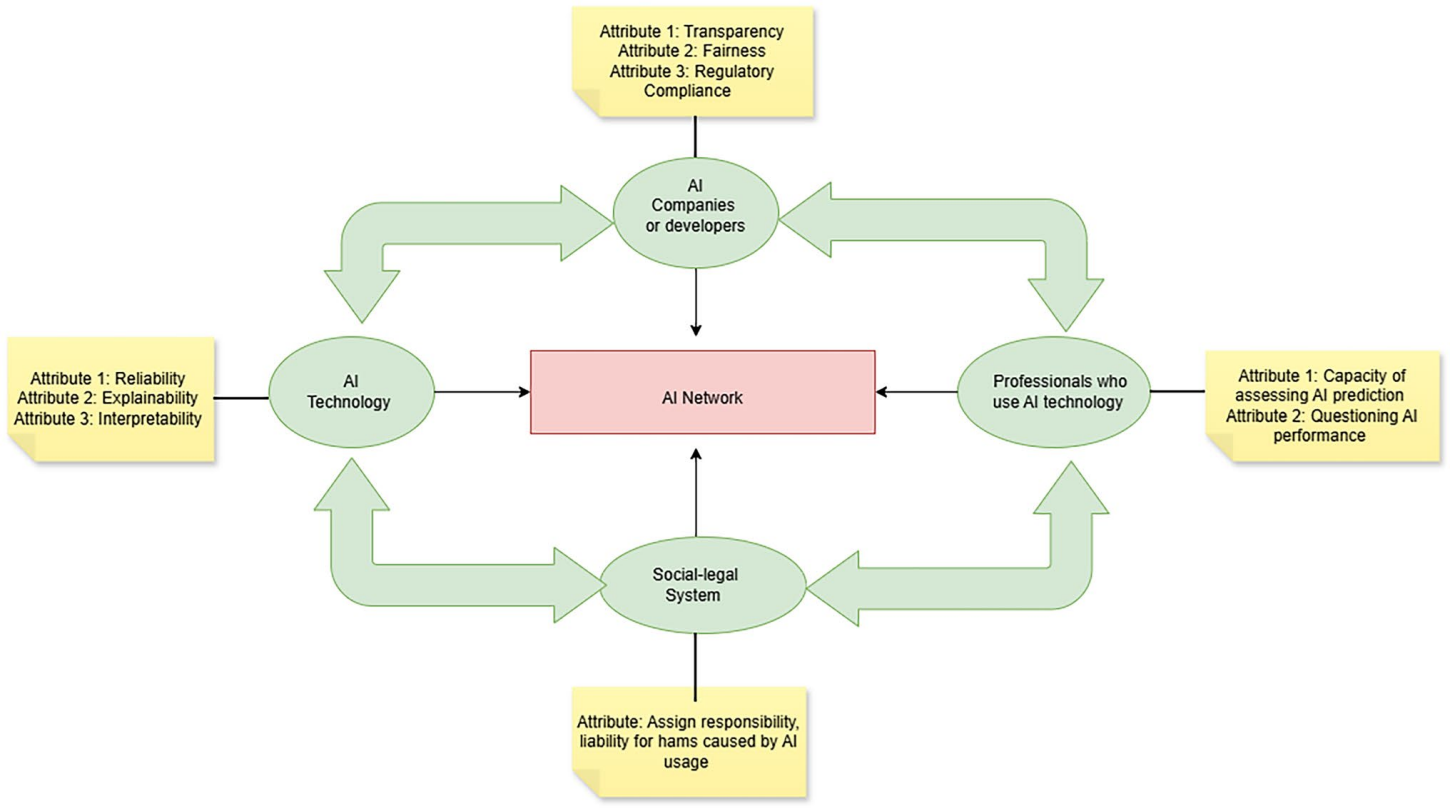
Network account of Trustworthy AI

Figure 1 illustrates the basic idea and structure of the network account of trustworthy AI:

### Ascertaining trustworthiness at the network level

How does each node in the system connect with others, and how do they function together to determine whether an AI system is trustworthy? In general, while in different contexts, the weights assigned to each node in assessing trustworthiness may vary, we may still be able to identify a common pattern.

First, we assert that trustworthiness is a matter of degree, meaning that an AI system can be considered maximally trustworthy if all the nodes within the system successfully secure their required attributes. In contrast, if an AI system is untrustworthy if all the nodes within the network are neither reliable nor trustworthy by reference to the criteria provided above. However, more realistic scenarios in the current use of AI in a social context are those in which the network is neither maximally trustworthy nor fully untrustworthy. Most commonly, we would expect some nodes to satisfy the required attributes, but some not to. The question then is what to make of trustworthiness in networks of these kinds. Consider two types of cases as such in a breast cancer detection AI context.

*Exceptional Case 1* One type of exceptional cases is that the AI technology itself demonstrates high reliability, producing accurate results in breast cancer detection across various demographics (say 95%), the surrounding nodes—such as the AI developers, physicians, and the legal system—are not fully trustworthy. There is a lack of clear commitment from AI developers to ensure fairness and transparency, as well as a failure to inform users about potential malfunctions or how to address errors. Furthermore, physicians often lack the necessary training and compacity to interpret AI results effectively, and the absence of a well-established legal framework for accountability and liability raises concerns about patient safety and the ethical use of AI in healthcare.

*Exceptional Case 2* In contrast, another type of exceptional case is that the AI algorithm used for detecting breast cancer at various stages produces less accurate results (e.g. < 70%). While there is good evidence that AI developers are committed to creating fair (unbiased) and just algorithms, as well as safeguarding personal data, although the results remain suboptimal. Meanwhile, the developers provide a thorough and transparent user manual, which clearly outlines potential problems that could lead to malfunction or incorrect results, and openly acknowledges the lack of solutions to some of these issues. However, physicians lack sufficient training to interpret or evaluate AI results due to the limited explainability of AI decision-making processes. Despite this, the social and legal systems provide well-established accountability and liability frameworks to address any risks or harms that may arise from the usage of the AI system.

#### Global assessment of trustworthiness

The most challenging question here is to provide a robust method for the global assessment of an AI network's trustworthiness, particularly a method that can effectively address the hard cases mentioned above.

*Aggregation approach*

One natural method is the aggregation approach, which is commonly used to address the challenge of decision-making criteria that are often numerous, interdependent, and sometimes conflicting. Let an AI network be represented as

$$N = \left\{ {N_{1} , N_{2} , \ldots N_{K} } \right\}$$

where each node $N_{i}$ represents a distinct node (e.g., AI technology, AI companies and developer) Each node has a set of attributes:

$$A_{i} = \left\{ {a_{i1} , a_{i2} , \ldots .a_{im} } \right\}$$

Corresponding to measurable or qualitative properties such as reliability, explainability, or accountability.

The aggregation approach assumes that the overall network value $V\left( N \right)$ can be computed as a weighted sum of normalized attributes,

$$V\left( N \right) = \mathop \sum \limits_{i - 1}^{k} w_{i } \cdot f\left( {A_{i} } \right)$$

where $w_{i }$ = inter-node weight, and $f\left( {A_{i} } \right)$ = a function of the total normalized value of attributes for each node $N_{i}$.

However, the aggregation approach faces challenges. First, the attributes of each node can be quantitative—typically numerical values derived from measurements or providing a comprehensive, statistical overview of a phenomenon—such as we can assess the reliability of the AI technology quantitatively by referring to, for example, the goodness-of-fit tests.^22^ Or some attributes can be qualitative—based on the detailed analysis and interpretation of a limited number of samples—such as the goodwill of the AI companies or the transparency of the reports. Once the relevant attributes are identified, aggregating them becomes complex due to issues of commensurability.

Second, even if we can identify a sound method for normalizing all attributes of the AI network onto a single scale, the next issue concerns how weight should be assigned to each attribute within the node and cross different nodes. The weight assigned to each attribute of a particular node may be highly subjective, and sensitive to both the specific context and the domain in which the AI system operates. A medical AI system, for example, might give higher weights to accuracy and reliability over explainability, whereas in legal or financial applications, transparency and accountability of the AI developers might be considered more critical. There lacks a universal weighting scheme that can be applied to all contexts.^23^

*Lexical ordering approach*

An alternative approach is the lexical ordering approach. The lexical ordering approach establishes a hierarchical relation among the attributes or nodes already defined in ${\mathcal{N}}$. Instead of aggregating values across attributes, this framework imposes a strict priority structure such that higher-order attributes cannot be compromised by gains in lower-order ones. Formally, we define a lexical priority relation $\succ_{L}$ over the attributes of the network:

$$N_{1} \succ_{L} N_{2} \succ_{L} \ldots . \succ_{L} N_{k} ,$$

meaning that the nodes themselves are ordered by priority — e.g.,

$${\text{AI Technology}} \succ_{L} {\mathrm{Developers}} \succ_{L} {\mathrm{Professionals}} \succ_{L} {\mathrm{Institutions}}{.}$$

Within each $N_{i}$, attributes are lexically ordered as:

$a_{1} \succ_{L} a_{2} \succ_{L} \cdots \succ_{L} a_{m} ,$ Attributes associated with higher-order nodes take precedence over those in lower-order nodes. Therefore, two AI networks, ${\mathcal{N}}_{A}$ and ${\mathcal{N}}_{B}$, are compared lexically as follows:

${\mathcal{N}}_{A} \succ_{L} {\mathcal{N}}_{B} {\text{ iff }}\widehat{ a}_{Apj } > \hat{a}_{Bpj} \; for \; the \;  first \; attribute \;  a_{pj} \; in \; the \; ranking \; where \; \hat{a}_{Apj } \ne \hat{a}_{Bpj}$ where $\hat{a}_{ij}$ denotes the normalized score of attributes $a_{ij}$ on the common $\left[ {0,1} \right]$ scale. This formulation means an AI network $N_{A}$ is judged superior to another network ${\mathcal{N}}_{B}$ if and only if (i) at the highest-order (inter-node) level, $A$ outperforms $B$ on the first node $N_{p}$ where their node-level evaluations differ; or (ii) If the two networks are equal on that node, preference is determined by the intra-node (attribute-level) lexical ordering.^24^

The lexical ordering approach does, however, faces a few changes. First, one basic difficulty concerns determining and agreeing on the priority ranking itself. In complex decisions, different stakeholders often disagree on what the “lexicographic” ordering should be. It may be straightforward in a personal choice for an individual to declare, for example, “I care about safety and reliability of an AI technology first, then the goodwill of the AI companies.” But in group/social decision-making, achieving consensus that one particular node/attribute is more important than the others, is hard.^25^

Even if a priority ranking is set, applying it consistently is often problematic. Lexical ordering works best when comparisons are clearcut and where one option unequivocally outperforms another on the top criterion (such as the maximally trustworthy case vs the maximally untrustworthy case). But correctly evaluating such criteria in practice will usually involve trade-offs. Consider Table 1. AI network $A$ may achieve a high level of reliability, yet professionals might lack sufficient training to operate the technology or interpret its results effectively. In contrast, AI system $B$ may be less reliable, but the company behind it demonstrates strong trustworthiness and a genuine commitment to user well-being. Lexicographic methods do not allow any compensating trade—so a tiny improvement on the top criterion can “trump” large losses on a secondary criterion. In practice, this can lead to counterintuitive or inefficient choices.^26^

*A better alternative: lexical threshold-based multidimensional utility approach* In this section, we propose a novel alternative, which we refer to as the *Lexical Threshold-based Multidimensional Utility Approach* (hereafter LTMU). The LTMU is a hybrid approach combining the idea of threshold-based lexical ordering with Martin Smith’s multidimensional utility theory (hereafter MUT) theory to address the dual problems of incommensurability and strict prioritization, associated with the two traditional approaches.

The hybrid LTMU approach works in *four* steps. In the first step, a lexical hierarchy is established over nodes and attributes:

$$N_{1} \succ_{L} N_{2} \succ_{L} \ldots . \succ_{L} N_{k} ,$$

$$a_{1} \succ_{L} a_{2} \succ_{L} \cdots \succ_{L} a_{m} ,$$

for example:

AI technology $\succ_{L}$ AI companies $\succ_{L}$ professionals; and Reliability $\succ_{L}$ Explainability.^27^

In the second step, we propose stipulating thresholds for each attribute to be considered sufficient. This stipulation is, first and foremost, context dependent—including domain (e.g., medicine) and culture (e.g., west and east). For each attribute $a_{j}$, a context-dependent sufficiency threshold $\tau_{j} \left( C \right)$ is defined such that:

$$\hat{a}_{j} \ge \tau_{j} \left( C \right) \Rightarrow a_{j} {\text{ is deemed sufficient}}{.}$$

Thresholds vary across domains $C$(e.g., medical, legal, financial). As we suggested in Sect. 4.1, for instance, in high-stakes domains—such as medical applications—it is reasonable to expect a significantly higher level of reliability (e.g., > 95%) compared to low-stakes contexts, such as résumé screening. Secondly, the thresholds may be somewhat arbitrary. We may not always be able to justify why, for example, 95% rather than 90% is chosen as the cutoff for sufficient reliability. However, we do not regard this arbitrariness as problematic, as it is quite common in all kinds of threshold-drawing theories. In statistics, for example, the selection of a p-value is often made by convention and widely accepted despite its arbitrary nature. Analogously, within specific domains, thresholds for sufficiency can be agreed through a collaborative and democratic process involving domain experts, AI developers, and regulatory bodies.

In the third step, following Smith’s MUT framework, each attribute $a_{j}$ is treated as an independent utility dimension, rather than being collapsed into a single value. Thus, the system’s performance is represented by a vector of utilities:

$U\left( {{\mathcal{N}},C} \right) = \left( {u_{1} \left( {\hat{a}_{1} } \right),u_{2} \left( {\hat{a}_{2} } \right), \ldots ,u_{m} \left( {\hat{a}_{m} } \right)} \right),$ where $u_{j}$ maps the normalized attribute score onto a domain-specific utility function. This move means that we do not need to determine ${\Phi }_{C} \left( {a_{ij} } \right)$ in the aggregation approach. Instead, we can assign distinct utility function to different attributes. For reliability, for example, small deviations near the top (0.95 → 0.90) may cause a sharp utility drop, so $u_{j}$ of reliability should be concave (e.g. $u_{R} ($​$\hat{a}_{R } ) = \hat{a}_{R}^{2} )$. For goodwill, it might be more linear (e.g. $u_{G} ($​$\hat{a}_{G } ) = \sqrt {\hat{a}_{G} } )$). For competence, it might be a linear proportionate function (e.g., $u_{C} ($​$\hat{a}_{C } ) = \hat{a}_{c} )$.

In the fourth step, if all high-priority attributes meet their threshold requirements:

$$\forall j \le J^{*} ,\;\hat{a}_{j} \ge \tau_{j} \left( C \right)$$

then the global trustworthiness value of the AI network is evaluated through a context-sensitive weighted aggregation:

$$V\left( {{\mathcal{N}},C} \right) = \mathop \sum \limits_{j = 1}^{m} u_{j} \left( {\hat{a}_{j} } \right), if \forall j \le J^{*} ,\;\hat{a}_{j} \ge \tau_{j} \left( C \right)$$

If any higher-tier attribute fails its threshold ($\hat{a}_{j} < \tau_{j} \left( C \right)$ for $j \le J^{*}$), the AI network is deemed below the threshold of trustworthiness, and lower-tier trade-offs are not considered. However, within the category of untrustworthy AI networks, some may be considered more untrustworthy than others, particularly if they fail to satisfy higher-tier attributes.^28^ The summary formula:^29^

$$V\left( {N,C} \right) = \left\{ {\begin{array}{*{20}c} {\mathop \sum \limits_{j = 1}^{m} u_{j} \left( {\hat{a}_{j} } \right), if \forall j \le J^{*} ,\;\hat{a}_{j} \ge \tau_{j} \left( C \right)} \\ {Untrustworthy, otherwise} \\ \end{array} } \right.$$

### Case Study

Reconsider the **Exceptional Case 1** and **Exceptional Case 2** presented in Table 1 and Table 2:

**Table 1:** Attribute﻿s

Node	Attribute 1 ($a_{i1}$)	Attribute 2($a_{i2}$)
$N_{1}$	Reliability	
$N_{2}$	Goodwill	Transparency
$N_{3}$	Competence	Accountability
$N_{4}$	Fairness	Liability/Governance

Within the LTMU framework, both Exceptional Case 1 and Exceptional Case 2 are deemed untrustworthy, as neither fulfils all threshold conditions. However, the network in Exceptional Case 2 is assessed as more untrustworthy than Exceptional Case 1, owing to its failure on reliability—the top-tier attribute in the lexical hierarchy—regardless of its performance on lower-ranked attributes.

The LTMU offers at least two key advantages: (1) it prevents excessive trade-offs, ensuring that a very low score in one attribute (e.g., reliability) cannot be fully compensated by exceptionally high scores in others; (2) it avoids a strict prohibition on trade-offs by allowing limited trade-offs once all attributes meet their minimum threshold values.

#### Objections and reply

The first objection is that **Exceptional Case 2** may, in fact, be better in some sense than** Exceptional Case 1**. This claim might be shared by many people. There are at least three possible explanations behind this intuition. First, the LTMU yields the correct result: **Exceptional Case 2** is in fact untrustworthy, but less so than **Exceptional Case 1**. By adjusting the parameters of the formula (e.g., $U\left( {N, C} \right)$), we would obtain the outcome that the network in **Exceptional Case 2** has a higher overall value than that in **Exceptional Case 1**. Second, the LTMU again yields the correct result: **Exceptional Case 2** is untrustworthy. However, our intuition here pertains not to trustworthiness but to permissibility—the AI network in **Exceptional Case 2** may still be permissible to implement. Developing a plausible account of permissibility, however, lies beyond the scope of this paper. Third, the LTMU is correct, and **Exceptional Case 2** is trustworthy under the following conditions:

- If developers clearly and transparently specify the limitations, accuracy range, and potential error modes of their systems, this allows the scope of legitimate application to be clearly delineated.
- Complemented by a well-designed liability regime that protects users from foreseeable harms, such systems—when deployed under suitable safeguards and with explicit constraints—could still be considered highly reliable within their defined operational domain.

The second objection concerns the rationale for adopting the LTMU approach instead of other alternative methods, such as Lexicographic max–min (LMM) (Ogryczak & Sliwiński 2006) or Choquet integral (CI) (Tversky and Kahneman 1992).^30^ We would respond, first, by arguing that the LMM is not a suitable candidate. Its core principle is to maximize the smallest objective value first; if the smallest values are equal across options, it then maximizes the second smallest, and so on in sequence. However, applying the LMM in the context of trustworthy AI can produce implausible results. For example, reconsider Table 2. The LMM suggests that $N_{A}$ performs better than $N_{B}$ because the attribute with the lowest priority—professional responsibility—has a slightly higher value for $N_{A}$(0.78) than for $N_{B}$(0.75). This outcome is counterintuitive: $N_{B}$ outperforms $N_{A}$ on all other dimensions except responsibility. Hence, while the LMM may be a reasonable approach for allocation problems, it is unsuitable for assessing an AI network’s overall trustworthiness.

**Table 2:** Exceptional Case 1 vs Exceptional Case 2

Attribute	$\hat{a}_{A}$	$\hat{a}_{B}$
Reliability	√	×
Goodwill	×	√
Transparency	×	√
Competence	×	√
Accountability	×	√
Fairness	×	√
Liability/Governance	×	√

In contrast, the Choquet integral might be more plausible than LMM. Under the Choquet integral method, we define a capacity $\mu$ to reflect perceived importance and independence between nodes. For example, (Table 3).

**Table 3:** Capacity measure table

Node Subset	$\mu \left( {Subset} \right)$
Θ	0
Tech	0.35
Dev	0.20
{Tech., Dev}	0.75

The table shows that $\mu$(Tech, Dev) > $\mu$(Tech) + $\mu$(Dev), meaning AI technology reliability and developer transparency jointly reinforce trust beyond simply addition. We can apply the same reasoning to the attributes. Is the Choquet integral a better candidate? The answer remains uncertain. The Choquet integral captures an important feature—the interdependence among nodes or attributes—which is not captured by other methods. However, it faces the same limitation as the aggregation approach: we lack a principled and justifiable method for determining the measure $\mu$.^31^ While we do not rule out the potential of the Choquet integral, further work is needed to examine the plausibility of this approach against the LTMU in future research.

## Conclusion

As AI technologies become increasingly integrated into our social, economic, and institutional structures, the question of what constitutes a trustworthy AI system becomes more urgent and complex. This paper has argued that existing frameworks for evaluating AI trustworthiness—many of which remain narrowly focused on algorithmic performance—are insufficient for capturing the full scope of H-AI trust relations. Building on Song’s (2023) network account, we argue that trustworthiness should be understood as an emergent property of a network system of technical, human, and institutional elements. To better operationalised this network-based account of trustworthiness, we put forward the *Lexical Threshold-Based Multidimensional Utility Theory*. By proposing a lexically ordered set of trust-relevant attributes, each with its own minimum threshold and evaluative metric, our approach offers a principled way to assess trustworthiness in complex AI networks—particularly in cases where attributes are in tension or appear incommensurable.

## Supplementary Information

Below is the link to the electronic supplementary material.

Supplementary file1 (DOCX 28 KB)

## Data Availability

No datasets were generated or analysed during the current study.

## Footnotes

1 A growing body of literature suggests that patient’s trust in medical practice extends beyond mere reliance. See (Weber 2024).

2 The four-levels of ethical agency are 1) Ethical Impact Agents: Entities whose actions have ethical consequences, regardless of intent. 2) Implicit Ethical Agents: Systems designed with safeguards to prevent unethical outcomes. 3) Explicit Ethical Agents: Agents capable of processing ethical principles to guide their actions. 4) Full Ethical Agents: Beings with consciousness and intentionality, enabling genuine moral reasoning. See (Tavani 2015, pp.77-78).

3 Although Zanotti et al. (2024) frequently emphasize that “AI systems are not ethically neutral” (p. 2696), it remains unclear whether the term “AI system” refers solely to the underlying technology or to a broader ecosystem that includes AI technologies, companies, human actors, and institutions. In presenting their new account of trustworthy AI, the authors argue that AI algorithms should not only be accurate, but also fair, transparent, and explainable (p. 2695). This framing suggests that the ethical requirements primarily apply to the algorithms themselves. However, they also highlight the importance of ethical considerations in the deployment of AI (p. 2692), implying that other stakeholders—such as AI companies—may also bear responsibility. Nevertheless, Zanotti et al. do not explicitly state that the assessment of AI trustworthiness should extend beyond the technology itself.

4 Due to space constraints, we provide only a brief overview of what we consider sufficient criteria for each node. A comprehensive analysis of these criteria would require a full research article.

5 The proposal of the four types of nodes refers to the document on ethical guidance for trustworthy AI proposed by European Commission’s High-level Expert Group on AI. Scholars suggest that AI has multi-characteristics: a) the AI technology itself; b) designers and organisations behind the development, deployment and use of AI; and c) the socio-technical systems involved in the AI life cycle (HLEG 2019, p. 5).

6 For the proposal and explanation of neuro-symbolic AI system, please refer to (Hitzler et al. 2022).

7 The general idea of statistical learning theory is like this: Consider a supervised learning model with a data distribution P and a space of candidacidal functions F. We draw a finite sample D of size N from P, and want to select a function f from F. The reliability refers to the predictive accuracy of model trained by the selected data D within in the data distribution P.

8 This approach tests the extent to which accuracy declines when the data distribution shifts. For example, consider a medical diagnosis model trained on data from Singapore. This approach aims to assess if the accuracy unreasonably drops when deployed in the context of Hong Kong. For more explanation of the statistical learning approach please refer to (Luxburg & Schoelkopf 2008).

9 This approach suggests that reliability should concern how model predictions are used and trusted by people, and how they integrate with human expertise and evidence evaluation.

10 The last approach focuses on the correctness of the internal reasoning of the model. To assess if the internal reasoning is flawed or not, it requires the results is interpretable or explainable. This approach seeks to open the black box by empirically analyzing network internals (borrowing techniques from neuroscience and experimental science) For more detailed illustration of explainable AI, please refer to (Adadi & Berrada 2018; London 2019; Rudin 2019).

11 Consider a medical diagnosis model that is trained by the early Covid-19 data. The virus is mutated fast and frequently, and thereby symptoms from the infections also changes. The nature of the virus, therefore, causes extreme difficulty to achieve the robustness. See more discussion from (Finlayson et al. 2021).

12 The “black box” problem refers to the problem of lacking interpretability and transparency inherited by the nature of the method of learning from big data. The explainability problem has spurred the field of explainable AI (XAI). The LIME (local interpretable model-agnostic explanations) and SHAP methods are the most dominate in the literature of XAI. LIME, for example, interprets a black-box model by generating perturbed samples around a given instance, weighting them by proximity, and training a simple, interpretable local model (e.g., a decision tree) to approximate and explain the model’s prediction for that instance. For details of different method of developing explainable AI, please refer to the overview by (Linardatos et al. 2020).

13 We acknowledge that there may be more than four attributes relevant to the trustworthiness of an AI company, such as value alignment and harm prevention. However, due to space constraints, we are unable to explore each attribute in detail here. A more comprehensive treatment will be reserved for a future paper.

14 For example, OpenAI’s 2023 technical report on GPT-4 provided no details on the model’s architecture, size, training data, or training methods, citing competitive and safety reasons. See report from https://cdn.openai.com/papers/gpt-4.pdf. By contrast, DeepMind, for example, has published many of its model architectures and research in academic venues, and it helped pioneer model documentation standards to disclose AI systems’ characteristics. See Slah et al. ((Slah 2025))., “An approach to technical AGI safety and Safety and Security”, https://storage.googleapis.com/deepmind-media/DeepMind.com/Blog/evaluating-potential-cybersecurity-threats-of-advanced-ai/An_Approach_to_Technical_AGI_Safety_Apr_2025.pdf

15 See OpenAI website https://openai.com/business-data/

16 A good analogy is the reports published by sovereign wealth funds. Some funds are much more transparent than others. For example, GIC (Singapore)’s report provides data in extremely details related to their investments, see (Linardatos et al. 2020). In contrast, the report from ADIA (Abu Dhabi), for example, does not provide enough key or substantial information (e.g., the strategy of their investments, where did they invest). see https://www.adia.ae/en/publications.

17 For a detailed summary, please refer to (H. Idrees 2024) URL: https://medium.com/@hassaanidrees7/ethics-and-bias-in-transformers-ensuring-fairness-in-ai-models-75a4a41b7222

18 The draft of EU AI Act can be found in https://artificialintelligenceact.eu/.

19 However, the real OpenAI’s CEO initially voiced that the *“current draft of the EU AI Act”* seemed like *“over-regulating”* and mused about withdrawing ChatGPT from Europe if unable to comply. Se Coulter & Mukherjee (2023, May 26), “OpenAI CEO’s threat to quit EU draws lawmaker backlash”, https://www.reuters.com/technology/openai-ceos-threat-quit-eu-draws-lawmaker-backlash-2023-05-25/

20 In the literature, this is called epistemic responsibility towards AI. See (Naeem & Hauser 2024).

21 For the discussion of legal concept of market share liability, please refer to ("Market Share Liability", 1981;2016; Fischer 1981; Sheffet 1983).

22 The goodness-of-fit test in statistic, is often used to test how the sample distribution fit the theoretical distribution. It can also provide an alternative method for measuring reliability. For instance, to assess the reliability of an AI technology X, we define a reliable AI technology as one that produces accuracy above 95%. After testing the AI technology, we find that it achieves 97% accuracy in the sample. The goodness-of-fit test can then be used to determine whether the hypothesis that X is reliable should be rejected.

23 To clarify these two challenges with greater precision, we develop additional formalizations. The complete formulations and proofs are presented in the Appendix.

24 An example is provided in Appendix to illustrate the lexical ordering approach.

25 This issue is analogous to the challenge posed by the weighted-sum approach in determining the appropriate weights for each variable. For further discussion, see the final paragraph of the section on the aggregation approach.

26 The challenges associated with general lexicographic priority in decision-making are well-documented in the literature. See (Lamont & Favor 2017; Şimşek 2021; Smith,^2025^.)

27 We do not claim that this is the only correct lexical ordering for a trustworthy AI network. For instance, we recognize that rankings may be domain-specific—what is appropriate for a medical AI system may differ significantly from what is suitable for a financial AI system.

28 While certain nodes may be ontologically incomparable, agents are nonetheless capable of forming preferences among them. Even when the entities in question differ categorically—much like comparing an apple with the Sun—individuals can still establish ordered preferences by evaluating the attributes of each. For instance, one might judge the Sun as preferable to the apple (Sun ≻ apple) on the grounds that life itself depends on the Sun’s existence. The proposed framework is designed to accommodate such forms of subjectivity and contextual sensitivity. It recognizes that comparability need not be objective to be practically meaningful: in real-world decision-making, agents routinely articulate and negotiate context-dependent preference orderings to guide rational choice.

29 While certain nodes may be ontologically incomparable, agents are nonetheless capable of forming preferences among them. Even when the entities in question differ categorically—much like comparing an apple with the Sun—individuals can still establish ordered preferences by evaluating the attributes of each. For instance, one might judge the Sun as preferable to the apple (Sun ≻ apple) on the grounds that life itself depends on the Sun’s existence. The proposed framework is designed to accommodate such forms of subjectivity and contextual sensitivity. It recognizes that comparability need not be objective to be practically meaningful: in real-world decision-making, agents routinely articulate and negotiate context-dependent preference orderings to guide rational choice.

30 We sincerely appreciate the reviewer’s insightful comment in raising this concern.

31 For detailed discussion of the problems, please refer to *Aggregation Approach* section.
