# Supplementary material for: Beyond the algorithm: rethinking the network account of trustworthy ai through lexical threshold-based multidimensional utility analysis
Source: AI Soc. 2026 Mar 4;41(6):5703–15. doi: 10.1007/s00146-026-02909-x (PMC13356064; doi:10.1007/s00146-026-02909-x)
Supplement: Supplementary file 1 — Supplementary file1 (DOCX 28 KB) [file 146_2026_2909_MOESM1_ESM.docx]

Appendix (Technical Details, Examples, and Formal Definitions)

Let an AI network be represented as

$$N=\left\{ N_{1}, N_{2}, \ldots N_{K} \right\}$$

Where each node $N_{i}$ represents a distinct node (e.g., AI technology, AI companies and developer) Each node has a set of attributes:

$$A_{i}=\{a_{i1}, a_{i2}, \ldots.a_{im}\}$$

Corresponding to measurable or qualitative properties such as reliability, explainability, or accountability.

1. Aggregation Approach

Challenge 1: issue of commensurability (Formalizations)

The aggregation approach assumes that overall network value $V\left( N \right)$ can be computed as a weighted sum of normalized attributes,

$$V\left( N \right)=\sum_{i-1}^{k} w_{i}\cdot f\left( A_{i} \right)$$

$f\left( A_{i} \right)$, it represents how the total normalized value of all attributes within each node is calculated:

$$f\left( A_{i} \right)= \sum_{j=1}^{m} b_{ij}\cdot\hat{a}_{ij}$$

Where $\hat{a}_{ij}$= is the normalized value of intra-node attributes within $N_{i}$, and $b_{ij}$ is the weight of the intra-node attributes within $N_{i}$ where $\sum_{j=1}^{m} b_{ij}=1$. Since each attribute is distinct, normalization is the process of transforming all attributes to one unified numerical scale.

$$\hat{a}_{ij}=\Phi\left( a_{ij} \right)$$

Where $a_{ij}$denotes the raw value of the given attribute *j* in node $N_{i}$;$\Phi$maps heterogeneous scales into a unified metric space, say $[0,1]$ (i.e., $\hat{a}_{ij}\boldsymbol{\in}\left[ \mathbf{0,1} \right]\mathbf{)}$

To explain this point, suppose that if $N_{i}$ is “AI technology,” $a_{i1}$is *reliability*, $a_{i2}$ is *explainability*. The reliability as a probability (0.85), explainability as a qualitative rating (“high”). The $a_{i1}$= 0.85, and $a_{i2}$=high. Suppose reliability scores range from 70% to 99% across systems. For a given system with 85% reliability:

$$\hat{a}_{ij}=\frac{a_{ij}-a_{\min}}{a_{\max}-a_{\min}}=\frac{0.85-0.70}{0.99-0.70}\approx0.52$$

For *explainability*, suppose high= 0.8, medium= 0.5, low= 0.2. So, $\hat{a}_{i2}$= 0.8.

However, the weighted aggregation approach faces challenges. First, defining such $\Phi$ remains **philosophically and methodologically underdetermined**. The example illustrated above relies on a largely arbitrary definition of $\Phi$. A higher-order theoretical framework is required to justify the normalization of such qualitatively distinct variables. However, such framework is unknown.

Challenge 2: Context-dependent (formalizations)

The context-based weighting scheme can be formulised as:

$$\text{ }b_{ij}=b_{ij}(C)$$

where $C$denotes the context or domain (medical, legal, financial). The formula denotes the context-dependent value of the $j^{th}$ attribute within the $i^{th}$node of the AI network. Since all attributes are normalized before aggregation or comparison, their context-sensitive normalized score then becomes:

$$\hat{a}_{ij}(C)=\Phi_{C}(a_{ij})$$

Where $\Phi_{C}$is the context-specific normalization function that transforms the raw attribute value into a comparable score within $[0,1]$, and $\hat{a}_{ij}\left( C \right)\in\left[ 0,1 \right],$this reflects how well the attribute satisfies its domain-specific criteria. When context is explicitly included, the overall evaluation of the network becomes:

$$V(\mathcal{N,}C)=\sum_{i=1}^{k} w_{i}(C)\cdot f(A_{i}(C))$$

with

$$f(A_{i}(C))=\sum_{j=1}^{m} b_{ij}(C)\cdot\hat{a}_{ij}(C)$$

Where $w_{i}(C)$= context-dependent weight for each node, $b_{ij}(C)$= intra-node attribute weight determined by context, and $\hat{a}_{ij}(C)$= normalized, context-specific attribute score.

To sum up, the summary formula of a weighted sum approach of global trustworthiness is

$$V\left( N,C \right)=\sum_{i=1}^{k} w_{i}(C)\sum_{j=1}^{m} b_{ij}(C)\Phi_{C}(a_{ij})$$

Unfortunately, the value of $V(\mathcal{N,}C)$is inherently indeterminate, given that both the normalization function$\Phi$and the $C$-parameterized functions are philosophically and methodologically underdefined.

1. Lexical Ordering Approach

Example to illustrate:

For example, the propriety order of the first order level is^[[1]](#footnote-1)^ :

$$\text{ }N_{1}\text{=AI Technology}; N_{2}\text{=Developers}; N_{3}\text{=Professionals}$$

Within each node, there are two attributes (second-order level):

| Node | Attribute 1 ($a_{i1}$) | Attribute 2($a_{i2}$) |
| --- | --- | --- |
| $N_{1}$ | Reliability | Explainability |
| $N_{2}$ | Goodwill | Transparency |
| $N_{3}$ | Competence | Responsibility |

Table 1 Attributes within node

We assume the lexical order is: ${N_{1}\succ}_{L}N_{2}\succ_{L}N_{3}$; and within each node ${a_{i1}\succ}_{L}a_{i2}$.
and the normalized scores are:

| Node | Attribute | $\hat{a}_{Aij}$ | $\hat{a}_{Bij}$ |
| --- | --- | --- | --- |
| $N_{1}$ | Reliability | 0.74 | 0.96 |
| $N_{1}$ | Explainability | 0.80 | 0.85 |
| $N_{2}$ | Goodwill | 0.85 | 0.90 |
| $N_{2}$ | Transparency | 0.70 | 0.75 |
| $N_{3}$ | Competence | 0.80 | 0.80 |
| $N_{3}$ | Responsibility | 0.78 | 0.75 |

Table 2. Normalised Score

Since, at the AI technology node, the reliability score of Network $B$ (0.96) exceeds that of Network $A$ (0.74), there is no need to examine the remaining attributes. Reliability belongs to the highest-priority node and is itself the highest-priority attribute within that node.

$$\mathcal{N}_{B}\succ_{L}\mathcal{N}_{A}$$

1. Lexical Threshold-based Multidimensional Utility Approach

Example to illustrate:

Suppose we have two AI network A and B; and we further stipulate the two-order lexical order is:

AI technology $\succ_{L}$ Developers $\succ_{L}$ Professional

Reliability $\succ_{L}$ Goodwill $\succ_{L}$ Competence

We further set a threshold in the context $C=$ medical AI, say, $\tau_{reliability}$= 0.95, $\tau_{Goodwill}$= 0.70; $\tau_{Competence}$ = 0.60.

The parameters of each attribute is

| Attributes | $\hat{a}_{A}$ | $\hat{a}_{B}$ | Threshold | $u_{j}(\hat{a}_{j})$ | $u_{j}(\hat{a}_{A})$ | $u_{j}(\hat{a}_{B})$ |
| --- | --- | --- | --- | --- | --- | --- |
| Reliability | 0.97 | 0.96 | √ | $\hat{a}_{R}^{2}$ | 0.94 | 0.92 |
| Goodwill | 0.75 | 0.70 | √ | $\sqrt{\hat{a}_{G}}$ | 0.86 | 0.83 |
| Competence | 0.70 | 0.85 | √ | $\hat{a}_{c}$ | 0.70 | 0.85 |

Table 3 Parameters of Attributes

Since all thresholds are satisfied, we aggregate:

$$V_{A}\left( N, C \right)=0.94+0.86+0.70=2.5$$

$$V_{B}\left( N,C \right)=0.92+0.83+0.85=2.6$$

So, $N_{B}\succ_{L}N_{A}.$

1. Reliability having the highest priority over other attributes is also suggested in the literature. See (Luxburg & Schoelkopf, 2008) [↑](#footnote-ref-1)
